# A High Resolution Genetic Map Anchoring Scaffolds of the Sequenced Watermelon Genome

**DOI:** 10.1371/journal.pone.0029453

**Published:** 2012-01-11

**Authors:** Yi Ren, Hong Zhao, Qinghe Kou, Jiao Jiang, Shaogui Guo, Haiying Zhang, Wenju Hou, Xiaohua Zou, Honghe Sun, Guoyi Gong, Amnon Levi, Yong Xu

**Affiliations:** 1 National Engineering Research Center for Vegetables, BAAFS, Beijing, China; 2 College of Life Science, Capital Normal University, Beijing, China; 3 U.S. Vegetable Laboratory, United States Department of Agriculture-Agricultural Research Service, Charleston, South Carolina, United States of America; University of Oxford, United Kingdom

## Abstract

As part of our ongoing efforts to sequence and map the watermelon (*Citrullus* spp.) genome, we have constructed a high density genetic linkage map. The map positioned 234 watermelon genome sequence scaffolds (an average size of 1.41 Mb) that cover about 330 Mb and account for 93.5% of the 353 Mb of the assembled genomic sequences of the elite Chinese watermelon line 97103 (*Citrullus lanatus* var. *lanatus*). The genetic map was constructed using an F_8_ population of 103 recombinant inbred lines (RILs). The RILs are derived from a cross between the line 97103 and the United States Plant Introduction (PI) 296341-FR (*C. lanatus* var. *citroides*) that contains resistance to fusarium wilt (races 0, 1, and 2). The genetic map consists of eleven linkage groups that include 698 simple sequence repeat (SSR), 219 insertion-deletion (InDel) and 36 structure variation (SV) markers and spans ∼800 cM with a mean marker interval of 0.8 cM. Using fluorescent *in situ* hybridization (FISH) with 11 BACs that produced chromosome-specifc signals, we have depicted watermelon chromosomes that correspond to the eleven linkage groups constructed in this study. The high resolution genetic map developed here should be a useful platform for the assembly of the watermelon genome, for the development of sequence-based markers used in breeding programs, and for the identification of genes associated with important agricultural traits.

## Introduction

Watermelon [*Citrullus lanatus* (Thunb.) Matsum & Nakai] is an important specialty crop accounting for ∼7% of the agricultural area devoted to vegetable crops (FAO, 2009). China is the largest producer and consumer of this crop, with an annual production of about 68 million tons (http://faostat.fao.org/site). Because of many years of cultivation and selection for watermelon of desirable qualities, the modern watermelon cultivars share a narrow genetic base, and are susceptible to a large number of diseases and pests [1]. There is a continuous need to develop new watermelon varieties with enhanced disease and pest resistance and with enhanced fruit quality, suitable to consumer needs. The watermelon includes several subspecies with a wide genetic diversity that could be a useful germplasm sources for improving watermelon cultivars [1]. Several critical steps that include accurate phenotyping for disease or pest resistance, genetic mapping and genome sequencing and assembly studies are needed as part of continuous efforts of exploring and utilizing watermelon germplasm for the improvement of watermelon cultivars. There are limited genomic resources and genetic maps for watermelon [2], [3], [4] and there is a need to develop a saturated genetic map that represent the watermelon genome. Also, there is a need for a cytogenetic map that could be useful in the identification of watermelon chromosomes and demarcating possible structural differences between the cultivated watermelon and it's related *Citrullus* spp.

Watermelon has a genome of 425 Mb (2n = 2x = 22 [5]). Several genetic linkage maps have been constructed for watermelon, in which markers (<400 loci) have often been positioned on more than 11 linkage groups with uneven distribution. These maps were mainly based on isozymes [6], [7], RAPD (randomly amplified polymorphic DNA), RFLP (restriction fragment length polymorphism), AFLP (amplified fragment length polymorphism) and SRAP (sequence related amplified polymorphism) markers, and only a limited number of SSR (simple sequence repeat) markers were used [4], [8]. The genetic maps were constructed using F2, BC1, or testcross populations [2], [9], [10], [11]. No high density genetic linkage maps representing genome sequences have been reported in watermelon, which has a low genetic diversity among different cultivars. Low polymorphisms have been discovered when using RAPD and SSR primers in watermelon genetic mapping experiments, while AFLP primers produced a higher rate of polymorphism. However, most AFLP markers were clustered on a few linkage groups and large regions of the watermelon genome could not be covered [4]. SSRs are abundant and ubiquitous in all eukaryotic genomes [12], [13]. They have become a valuable source of molecular markers for genetic analysis [14]. Only a few SSR markers (<100) have been developed for watermelon, mainly from expressed sequenced tag (EST) sequences of the watermelon fruit and from screening genomic libraries [4], [8]. Because of insufficient genome sequencing data, insertion and deletion (InDel) polymorphisms have received relatively little attention in watermelon. The structure variation (SV) markers may represent deletion, duplication, insertion, translocation or inversion of DNA segments in the genome, and may profoundly affect the correlation between genetic and physical distance for the same interval in plants [15]. No InDel or SV markers have been previously developed in watermelon.

As part of the International Watermelon Genome Initiative, we have initiated the watermelon genome sequencing project at the end of 2008 [16]. The resulting large dataset and the draft genome sequence provided sufficient resources to construct the highly saturated genetic map for watermelon. The SSRs, InDels, and SVs, which are sequence based and co-dominant markers, are ideal for anchoring and orienting watermelon assembled genome scaffolds.

The watermelon chromosomes are relatively small in size and are morphologically similar. They have not been extensively studied using fluorescent *in situ* hybridization (FISH) technologies. The most commonly used FISH probes for karyotyping in plant species are 5S and 45S rDNAs, tandemly repeated sequences near telomeres, and centromere-specific repeats. Their position and fluorescence intensity allow quick identification of certain somatic metaphase chromosomes. However, 5S and 45S rDNAs could not differentiate all eleven chromosomes. In this regard, using bacterial artificial chromosomes (BACs) probes as cytogenetic markers for FISH could be an effective approach to differentiate individual chromosomes.

Here, we report the development of a large number of SSR, InDel and SV markers, resulting from the genomic sequencing of the elite Chinese line 97103 and re-sequencing of wild accession PI 296341-FR. These markers were then used to create a high resolution genetic map that comprised of 11 linkage groups, which were further assigned to the corresponding 11 individual chromosomes using FISH. The genetic map was then used to assist in anchoring and orienting assembled genome scaffolds.

## Materials and Methods

### Plant materials

**Figure 1:**
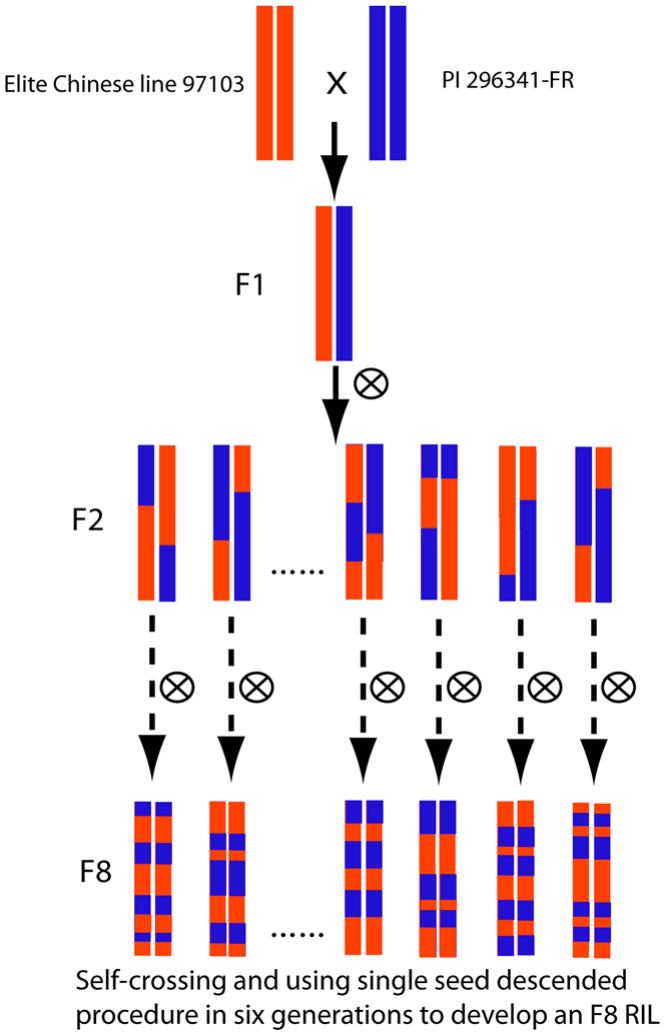
Construction of the RILs population.

An F_8_ population consisting of 103 recombinant inbred lines (RILs) was derived from a cross between the elite Chinese line 97103 (*C. lanatus* var. *lanatus*) and the U.S. Plant Introduction (PI) 296341-FR (*C. lanatus* var. *citroides*) (Figure 1). Total DNA was isolated from expanding leaves of three-week old plants using the modified CTAB method [17]. Genomes of the two parents were sequenced using the next-generation Illumina GA sequencing technology (Illumina, Inc., San Diego, CA, USA). Illumina sequences of the line 97103 genome were assembled into contigs and scaffolds using the method described in Huang et al. [18].

### Marker development

SSR, InDel and SV markers were developed from the assembled scaffolds of the line 97103 genome and genome sequence reads of PI 296341-FR. The line 97103 genome assembly had a total length of 353 Mb and contained 184 N90 scaffolds (N90 scaffolds refer to the largest scaffolds that in total cover 90% of the total assembled sequences). This assembly was used as the reference genome. The watermelon genome assembly is available at http://www.iwgi.org and http://www.icugi.org. PI 296341-FR, which is a wild accession of *Citrullus lanatus* var. *citroides*, was used for genome re-sequencing. In total, 4,996 Mb raw sequences were obtained and 3,958 Mb (9-fold coverage of the PI 296341-FR genome) could be mapped to the genome assembly of line 97103. Approximately 92% of the assembled 353 Mb line 97103 genome could be covered by PI 296341-FR Illumina reads with a mode depth of 9.0.

**Figure 2:**
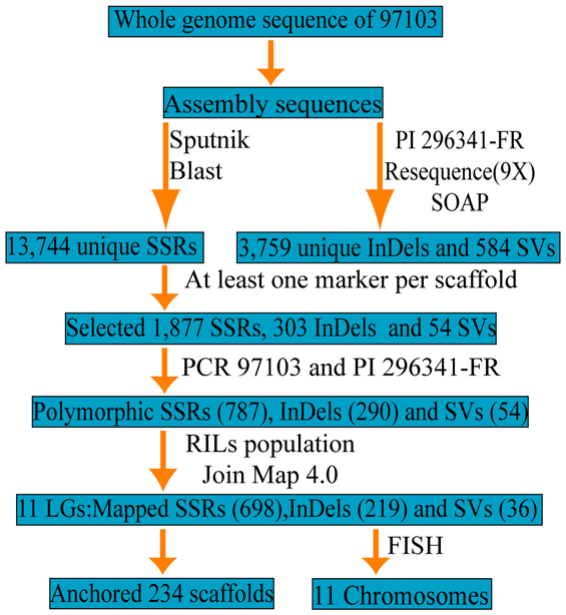
A schematic diagram of the development of watermelon SSR, InDel and SV markers.

The process of developing SSR, InDel and SV markers employed herein is presented in Figure 2. SSR markers were developed from the assembled scaffold sequences of line 97103, the InDel and SV markers were discovered by comparing genome sequences of line 97103 and PI 296341-FR. If a scaffold contained all three types of markers, SSR markers were chosen first for genetic mapping, then InDel and SV markers, until at least one polymorphic marker was found in this scaffold. For scaffolds that only contained InDel and SV loci, polymorphic InDel markers were selected preferentially. If a scaffold contained more than one marker, markers were chosen according to their position, such that they were distributed evenly in the scaffolds to avoid mapping redundant markers.

**SSR** In the genome sequences of line 97103, repeat motifs with lengths of at least 20bp were identified using a modified Sputnik program (modification specified by C. Abajian, http://espressosoftware.com/sputnik). The minimum repeat number was designated as three, and each putative SSR locus was defined as the region containing the SSR motif and 200 bp up- and downstream of the motif. All putative loci were compared to scaffolds by BLAST search at an E-value cutoff of 10^−40^. Loci with single hits were defined as unique loci and were used in map construction.

**InDel** InDel sites were identified by aligning PI296341-FR genome sequence reads to the line 97103 genome assembly with SOAPindel (http://soap.genomics.org.cn/). Each pair of InDel sites that co-located only within 5–10 bps was selected. Only InDels with single hits in a whole genome BLAST search at an E-value cutoff of 10^−40^ were used in map construction. False priming events in the watermelon genome were then identified with in-house perl scripts that can randomly choose a 13–15 bp sequence from the primer to detect the non-unique amplification. Only primers with one hit in the genome assembly were used.

**SV** Potential structure variation loci were identified by aligning PI296341-FR paired-end reads to the line 97103 genome assembly using SOAPsv (http://soap.genomics.org.cn/). Sizes of identified varied structures were restricted to 120–1400 bp, which fit our normal procedures for PCR and electrophoresis. Occurrences of false priming were checked in the same manner as that for InDel primers.

Primer pairs for identified SSR, InDel, and SV markers were designed using the Primer 3.0 program [19], and subsequently synthesized by Augct Biological Company, Beijing, China.

### PCR amplification and marker scoring

Polymerase chain reactions (PCR) were performed in 15 µl volumes containing approximately 20 ng template DNA, 1× buffer, 0.5 units Taq polymerase, 20 ng of forward and reverse primers, 2 mM dNTPs. Optimized PCR thermocycling for SSR and InDel markers incorporated a denaturation step of 5 min at 94°, followed by 35 cycles of 20 sec at 94°, 20 sec at 55°, 30 sec at 72°, and ended with a final 4 min extension step at 72°. PCR for SV markers employed a denaturation step of 5 min at 94°, followed by 35 cycles of 20 sec at 94°, 20 sec at 55°, 90 sec at 72°, and stopped after a final 8 min extension at 72°. Subsequently, 3 µl of the PCR product was used for electrophoresis in a 6% polyacrylamide gel using an optimized procedure [20]. Markers that were unique to line 97103 or PI 296341-FR and present in the F1 population, were used for genetic mapping using the population of 103 RILs.

### Genetic map construction

After markers were visually scored, unambiguous markers were used for map construction, employing the JoinMap program version 4.0 [21]. Linkage groups (LGs) were identified with likelihood odd (LOD) ratios ≥10.0, and marker locations on LGs were graphically displayed using Microsoft Excel via a conditional cell formatting formula and points of disagreement designated as “singletons” were resolved by reassessment of band morphotypes. Singletons were deleted and co-segregating markers were used to infer “bin signatures” [22]. A bin signature comprises the consensus segregation pattern of marker loci, which do not recombine and are thus incorporated in the bin as described in Ren et al [23]. The resulting bins collectively allowed for the construction of a skeleton bin LG map that was then used as a reference map to calculate genetic distances and permitted filling of bins with marker loci for the development of the final map, which was constructed based on the reference map. Markers not located on the reference map were added by decreasing the LOD threshold in JoinMap with minimum LOD score set to 4. Only the marker sequences with unique hits in the genome sequence were included. The bins were numbered consecutively, thus resulting in a skeleton bin map. The consensus genotype data for bins were then used to calculate genetic distances.

In order to fill gaps in the genetic map, 87 RAPD [24] and 150 AFLP [25] markers from our previously published maps derived from the same RILs were used. Herein, the linkage groups were identified at lower LOD scores of 3.0 and 4.0 for LG1 and LG5, respectively. The segregation ratios of markers in the population were examined by Chi-square analysis. Markers with segregation ratios that differed from expected 1∶1 at P<0.05 were classified as segregation distortion markers. A region with ten or more adjacent loci showing significantly skewed segregation was defined as a segregation distortion region (SDR).

### Estimation of recombination rates

According to positions and orientation of anchored scaffolds, we assembled them into eleven pseudochromosomes corresponding to the eleven LGs. Each pseudochromosome was divided into non-overlapping 1-Mb segments, the starting and ending positions of which (in Mb) were determined based on the line 97103 reference genome. The genetic starting and ending positions (in cM) of segments were estimated based on the genetic map according to corresponding markers. The estimated recombination rate of a segment (cM/Mb) was calculated using in-house perl scripts, which divided the genetic length of the segment in cM by the physical length of the segment in Mb. We defined recombination suppression regions as where recombination rate was less than 1.0 cM/Mb and recombination hot regions as where recombination rate was more than 1.0 cM/Mb.

### BAC, 5S and 45S rDNA probe preparation for FISH

The BAC library reported in Joobeur et al [26] was constructed from the inbred line 97103 that was used for our genome sequencing. BAC clones for FISH analysis were kindly donated by Syngenta Company. We sequenced both ends of 947 BACs by Sanger sequencing technology (Applied Biosystems 3730xl DNA Analyzer) and produced 1664 high quality BAC end sequences. BAC end sequences were aligned to line 97103 genome scaffolds and only BACs with two ends aligned to the scaffolds properly were kept. The two end sequences from a BAC were mapped to the scaffolds properly if 1) one end was aligned in forward direction, while the other end in reverse direction, and 2) the distance between the two aligned positions on the reference scaffold should be in accordance with the estimated BAC size. Subsequently, sequences in the scaffold between the two aligned positions were extracted and used as the predicted BAC sequences. A whole genome BLAST search was conducted with the predicted BAC sequences and only BACs having sequences that were likely a single copy in the genome were selected as candidate probes for FISH. However, BACs located at the middle of linkage groups were not chosen for FISH, because repeat sequences may be abundant in the pericentromeric regions, which may result in multiple FISH signals. BAC DNA was extracted according to a standard procedure [20]. Analysis by FISH was performed according to Han et al [27], where BAC probes were labeled with either biotin-dUTP or digoxigenin-dUTP (Roche) via nick translation and detected with anti-digoxigenin antibody coupled with Rhodamine (Roche) or anti-avidin antibody conjugated with FITC (Vector Laboratories), respectively.

The watermelon 5S rDNA was amplified by PCR according to the study of Udovicic et al [28] using the following primer sets: 5′-CACCGGATCCCATCAGAACT-3′ (reverse) and 5′-TTAGTCTGGTATGATCGCA-3′ (forward). The purified 5S rDNA probe was labeled by PCR DIG Labeling Mixplus (Roche Diagnostics GmbH, Penzberg, Germany). The thermal cycle conditions were as follows: 95°C for 2 min, 30 cycles of 95°C for 45 s, 60°C for 1 min, and 72°C for 2 min, followed by 72°C for 10 min. Plasmid pTA71 which contains approximately 9 kb of the 45S rRNA repeat units of *Triticum aestivum* was used as 45S template DNA in this study [29]. Plasmid pTA71 was extracted using TIANpure Midi Plasmid Kit (TianGen Biotech) and then labeled with biotin-16-dUTP by nick-translation (Roche Diagnostics GmbH, Penzberg, Germany).

### Florescent *in situ* hybridization

Chromosome preparations were made according to Han et al [27]. Root tips were harvested from germinated seeds, pretreated at 4°C for 2–4 h to capture pro-metaphase and metaphase cells, and fixed in Carnoy's solution (3 ethanol: 1 glacial acetic acid). Root tips were then macerated in 2% cellulase and 1% pectolyase (Sigma, USA) at 37°C for 2 h, and squashes were prepared using the same fixative. Chromosomes were then counterstained using 4, 6-diamidino-2-phenylindole (DAPI) in the Vectashield antifade solution H-1200 (Vector Laboratories, Burlingame, CA). Slides were examined under a Nikon 80i photomicroscope equipped with epifluorescence illumination and filter sets for DAPI, FITC and Texas-Red fluorescence. At least 50 metaphase samples were examined for hybridization. Selected images were acquired using a digital camera (DS-2MBWc, Nikon, Japan) and were adjusted with Adobe Photoshop v 6.0 software.

## Results

### SSR, InDel, and SV marker development and polymorphism

SSR, InDel, and SV marker development was based on the assembled 353 Mb genome scaffold sequences of line 97103 and re-sequencing data of PI 296341-FR. A total of 13,744 putative SSR loci were identified. After eliminating SSR loci with more than one homology, 1,877 unique SSRs with long repeat motifs were selected for polymorphism analysis with the parental lines (97103 and PI 296341-FR) of the RIL population. Of these 1,877 SSRs, 787 (41.9%) showed polymorphism between line 97103 and PI 296341-FR after PCR amplification.

After choosing only InDel loci located within 5–10 bp and SVs from 120 bp to 1500 bp and eliminating loci with more than one homology, 3,759 InDels and 584 SVs were obtained. 303 InDels and 54 SVs were selected for polymorphism analysis between the two RIL parents (line 97103 and PI 296341-FR). Two hundred ninety (95.7%) of the 303 InDels and all 54 SVs were polymorphic between the parents.

### High resolution genetic map

**Figure 3:**
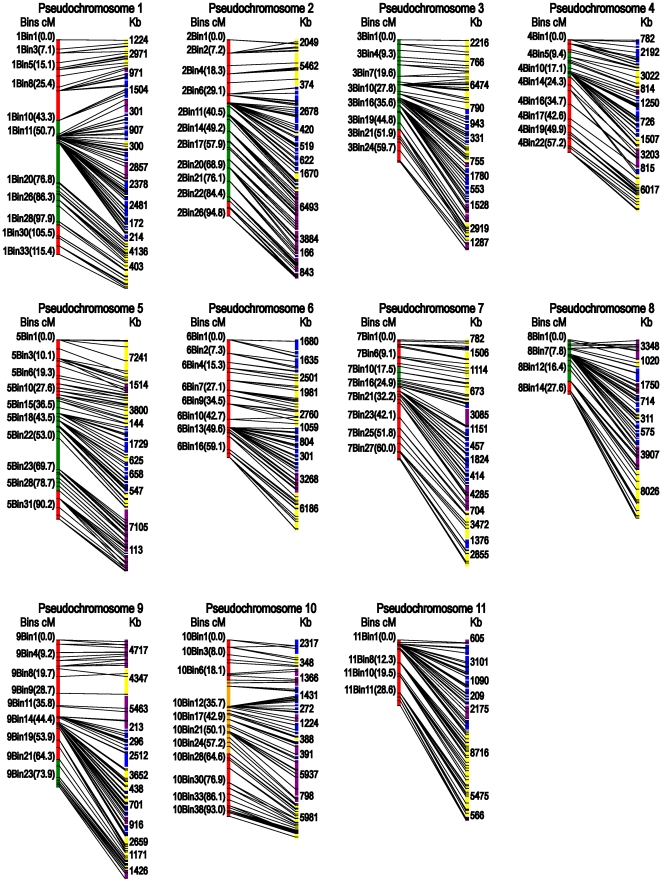
A high resolution linkage map for anchoring and orienting watermelon genome scaffolds. Bins and genetic distances in cM are listed on the left, scaffold lengths in Kb are listed on the right. Yellow scaffolds were oriented in forward direction, purple in reverse direction, and blue not oriented. Segregation distortion regions skewed toward line 97103 are labeled green and those skewed toward PI 296341-FR are orange.

Overall, a total of 953 markers (698 SSRs, 219 InDels and 36 SVs) were mapped in the RIL population, spanning ∼800 cM with a mean marker interval of 0.8 cM. Based on the estimated size of the watermelon genome (425 Mbp), the map defined herein represents average physical intervals of ∼450 Kb per marker, making it the most saturated map in watermelon to date. Around 640 recombination events (bins) were identified, among which 285 (44%) were filled with one or more markers (Table 1, Figure 3). The map consisted of eleven linkage groups, with the largest one containing 114 markers and spanning 115.4 cM and the smallest one containing only 63 loci and spanning 29.2 cM. Although genetic distances were quite variable among the eleven linkage groups, the corresponding physical distances ranged between 24.3 Mb and 35 Mb. In this map two large gaps between 1bin19 and 1bin20 (21.1 cM), and between 5bin22 and 5bin23 (16.7 cM) in LG1 and LG5, respectively, were detected (Figure 3). These two gaps are between scaffold 161 and scaffold 730 in LG1, and between scaffold 1 and scaffold 108 in LG5 (Table S1). Among 87 RAPD [24] and 150 AFLP [25] markers from our previous released maps derived from the same RILs, two RAPD markers were located between 1bin19 and 1bin20 and five AFLP markers could be used to fill another gap (5bin22–5bin23). Detailed information for the mapped SSR, InDel, SV markers and corresponding scaffolds is listed in Table S1.

**Table 1:** Summary of the watermelon genetic map generated from line 97103× PI 296341-FR recombinant inbred lines.

LGs	No.loci	No. anchoredscaffolds	Scaffold size(Mb)	Genetic distance(cM)	Recombinationevents	Filledbins
LG1	114	30	34.1	115.4	65	33
LG 2	107	23	34.4	94.8	57	26
LG 3	90	25	28.9	65.2	60	26
LG 4	71	18	24.3	60.5	54	24
LG 5	98	21	33.7	96.4	88	33
LG 6	79	20	27	63.3	51	18
LG 7	74	26	31.5	63.9	55	30
LG 8	63	16	26.1	29.2	28	15
LG 9	91	21	35	79.1	76	27
LG 10	91	20	28.4	94.9	85	39
LG 11	75	14	27.1	35.3	24	14
Total	953	234	330.4	798.0	643	285

Segregation distortion is prevalent in wide-cross populations, and plays a dominant part in plant genome evolution [30]. Nine segregation distortion regions (SDRs) were detected in LGs 1, 2, 3, 4, 5, 7, 8, 9 and 10, respectively (P<0.05). The SDRs in LGs 1, 2, 3, 5 and 8, spanned relatively large parts of the whole LG, from 43.3 to 99.5 cM, 33.9 to 86.9 cM, 0 to 48.3 cM, 30.9 to 81.3 cM and 0 to 22.3 cM, respectively (Figure 3). All marker alleles within these SDRs were associated with the *Citrullus lanatus* var. *lanatus* parent (line 97103) except those markers in LG 10 were skewed toward the *Citrullus lanatus* var. *citroides* parent (PI 296341-FR).

**Figure 4:**
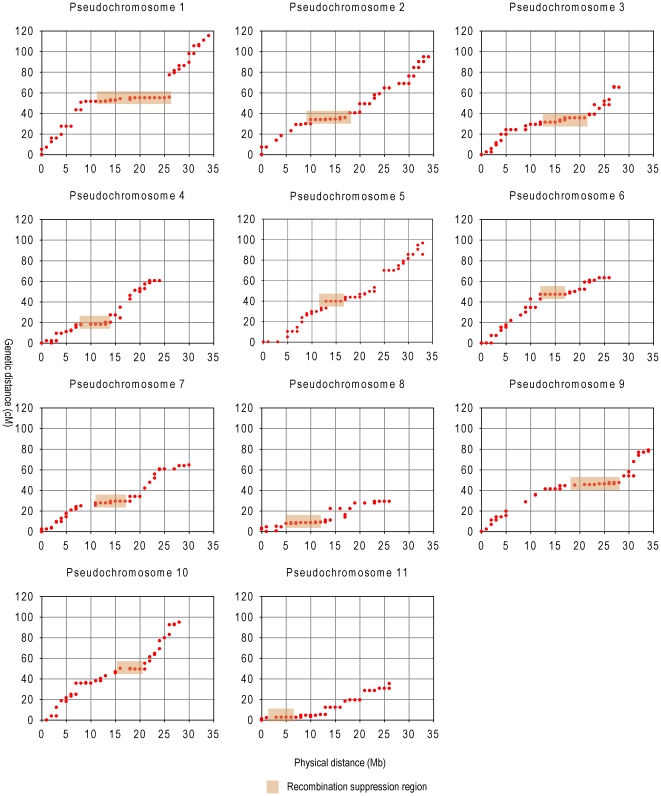
Plots of genetic vs physical distance for each linkage group. In each plot, physical distance is on the horizontal axis (in Mb), and genetic distance is on the vertical axis (in cM). The recombination suppression regions (nearly flat in shadow) are predicted to be pericentromeric regions.

Rate of recombination is the most important factor for genetic analysis, and holds numerous implications for both academic and applied realms. Recombination suppression and consistently high recombination at chromosome ends were obviously observed (Figure 4). We found eleven recombination suppression regions in the eleven LGs (Figure 4 and Table S1). Regions at chromosome ends consistently had higher recombination rates. We found that the recombination rates in hotspots regions ranged from 3.0 to 8.3 cM/Mb, while in the suppression regions, rates ranged from 0 to 0.9 cM/Mb. The average ratio of genetic-to-physical distance was 2.3 cM/Mb for the whole genome. This ratio is similar to those for cucumber, 3.2 cM/Mb [18] (this statistic was recalculated from the publication to better match the one presented in this paper, rather than presenting the number from the publication), and sorghum, 1.43 cM/Mb [31], but lower than that for maize, 5.5 cM/Mb [32].

### Scaffold anchoring and orientation

Scaffold placements were determined based on the high-resolution line 97103×PI296341-FR genetic map constructed in the present study which consisted of 953 markers. All together, 234 scaffolds totaling 330 Mb, accounting for 93.5% of the assembled 353 Mb sequences of the line 97103 genome were successfully anchored (Table 1), and 195 scaffolds were anchored by at least two different markers. On average, four markers were located in each scaffold. Ninety-four of the scaffolds (228.3 Mb; 64.7%) that had more than one marker could be oriented with high confidence on the genetic map (Table S1). For the 140 smaller scaffolds (each less than 1 Mb and a total of ∼70 Mb) that had only one genetic marker or were located in a genomic region with low recombination frequency, their orientation on the map was determined solely by chance. In summary, we assembled the scaffolds into eleven chromosomes, numbered according to the linkage group nomenclature. Detailed information for the anchored and oriented scaffolds is listed in Table S1.

### Assigning linkage groups to chromosomes

**Figure 5:**
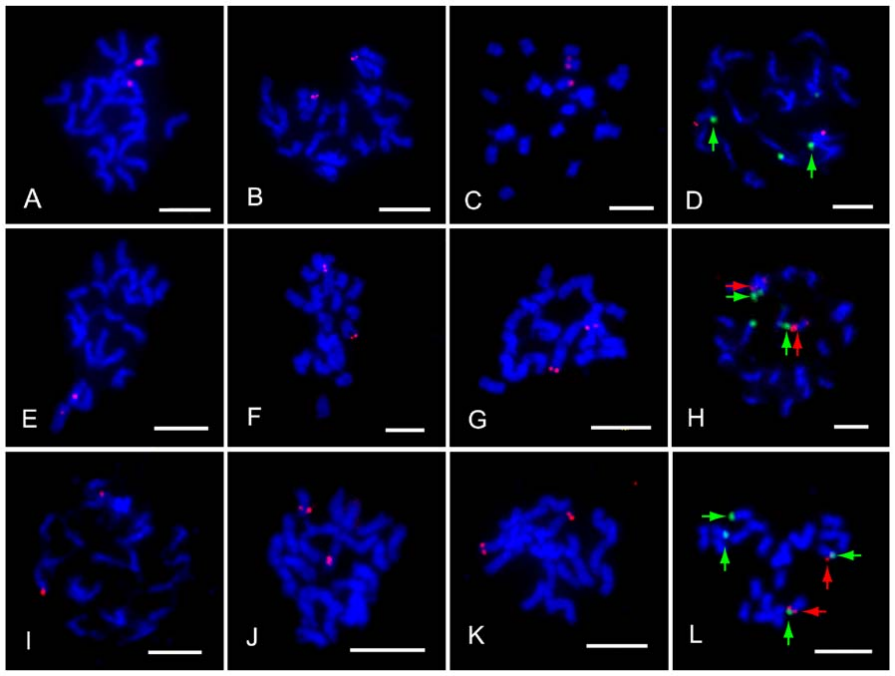
Assignment of linkage groups to eleven chromosomes. Arrowheads are locations of 45S (green) and 5S (red) rDNA sites in photographs D, H and L. Red signals are locations of BACs on each chromosome and 5S rDNA on chromosome 8. (A) to (C) Hybridization of BAC clones 10K19, 60M17 and 51B14 on chromosome 1, 2 and 3, respectively. (D) Hybridization of BAC clone 51N03 (red) and 45S rDNA (green) on chromosome 4. (E) to (G) Hybridization of BAC clones 109C21, 10M24 and 51P08 on chromosome 5, 6 and 7, respectively. (H) Hybridization of BAC clone 51F09 (red) and 5S rDNA (red arrowheads) and 45S rDNA (green arrowheads) on chromosome 8. (I) to (K) Hybridization of BAC clones 51A03, 36G07, 50J01 on chromosome 9, 10 and 11, respectively. (L) FISH pattern on the prometaphase chromosomes using both 5S (red) and 45S rDNA (green) probes. Scale bar = 5 µm.

We sequenced both ends of 947 BAC clones and obtained a total of 1664 high quality end sequences, which were further aligned to watermelon genome scaffolds. Based on the alignments, eighty six single-copy BACs were identified as candidate probes for FISH. Eleven of them that produced a chromosome-specific hybridization signal constantly during our FISH experiments were used to assign LGs to their corresponding chromosomes (Table S2 and Figure 5).

We also conducted FISH-mapping of 45S and 5S rDNA sites on mitotic chromosomes to identify chromosomes 4 and 8. We found that there were two 45S rDNA sites and one 5S rDNA site in the line 97103 genome. The 5S rDNA site was located syntenic to one of the 45S rDNA sites on chromosome 8, while another 45S rDNA site was located on chromosome 4 (Figure 5). Using this strategy, 45S rDNA, 5S rDNA and the 11 BAC clones that defined a single locus in the genome were assigned to corresponding chromosomes, allowing for the integration of each of the 11 linkage groups into corresponding chromosomes. For example we defined chromosome 1 as the chromosome which can hybridized with the single-copy BAC 10K19 that was located on scaffold619 within LG 1. The chromosome which can hybridized with BAC 51N03 located on scaffold170 within LG 4 and 45S rDNA located on scaffold25 within LG 4 was chromosome 4, and so on (Figure 5). Detailed information about the 11 chromosome-specific BACs and 45S and 5S rDNA markers for chromosome definition is listed in Table S2. Here we initially distinguish the 11 chromosomes by the 11 single-copy BACs, 45S rDNA and 5S rDNA rather than traditional karyotype analysis and assign eleven LGs to corresponding chromosomes. Furthermore, the 11 chromosome-specific BAC clones can also serve as reliable cytological markers in future cytogenetic studies of watermelon.

## Discussion

We described the construction of a watermelon genetic map utilizing polymorphic SSR, InDel and SV markers derived from whole genome sequencing and re-sequencing, including scaffold anchoring and orientation. This represents the first high resolution watermelon genetic map anchored with assembled genome scaffolds. The high density genetic map was used as the reference to assign linkage groups to corresponding chromosomes using FISH. This map provided a basis for anchoring and orienting the genome sequences and helped validate the integrity of the existing genome assembly. It is also valuable for future quantitative trait loci (QTL) mapping, molecular marker assisted breeding and positional gene cloning.

### Marker distribution and segregation distortion

In non-repetitive euchromatic regions, the repeat densities are lower and unique primers are more easily identified [33]. Consequently, the majority of SSR, InDel and SV markers were likely developed from non-repetitive regions of the genome, thus were likely associated predominantly with the euchromatic or gene-rich regions. This high density genetic map anchored ∼330 Mb, accounting for 93.5% of the assembled 353 Mb sequences. Markers were chosen to be evenly distributed in the scaffolds, providing better estimates of recombination across the whole genome, compared to previously reported watermelon genetic maps. According to results obtained for the cucumber genome [18], a majority of the remaining 30% of unassembled regions of the cucumber genome are likely to be heterochromatic satellite or rRNA sequences. In watermelon, we expected the same situation. It might be hypothesized that these markers were saturated in the non-repetitive or euchromatic regions of the genome, and the unassembled residual sequences (18% of whole genome) were mainly heterochromatic repeat sequences.

Two large gaps between 1bin19 and 1bin20 (21.1 cM) in LG1, and between 5bin22 and 5bin23 (16.7 cM) in LG5 were detected. The RAPD and AFLP markers used to fill these two gaps provided evidence that regions flanking the gap may be linked. However, compared to other markers, RAPD and AFLP markers are less reliable for genetic mapping, so additional SSR, InDel or SV markers within these regions need to be developed for the purpose of confirmation. Markers used for mapping were derived from the assembled scaffolds thus no markers existed for the unassembled sequences. However, random markers like RAPD and AFLP were detected in these gaps. Thus, these gaps might represent unassembled regions of the genome that were likely to be heterochromatic repeat sequences. Since these RAPD and AFLP markers did not contribute to our scaffold anchoring, they were not merged in this genetic map.

We found nine SDRs and eight of them were associated with the *Citrullus lanatus* var. *lanatus* parent (line 97103). A possible reason for this skewing is that genes in these regions in the elite parent, compared to the wild parent, may be associated with fruit and seed production, especially ease of production, characters that were undoubtedly under selection during the creation of the RILs population. Segregation distortion has been reported in watermelon genetic populations derived from a cross between a watermelon cultivars (*Citrullus lanatus* var. *lanatus*) and plant introduction accessions of *Citrullus lanatus* var. *citroides* [2], [4]. This preferential segregation for most markers could be the result of the wide genetic distance between the parents.

### Recombination suppression and high recombination

Recombination is a crucial component of evolution and breeding, leading to either the loss or gain of intergenic combinations in the recombinants, on which selection can act. However, recombination rate varies tremendously, not only among species but also within species and specific chromosomal segments. In this study, we found lower recombination rates in broad pericentromeric regions and consistent high recombination at ends in all 11 chromosomes.

We calculated the frequency of whole genome SNP loci of the two parents and found the SNP density was 7.14 SNP/kb. We calculated the distribution of SNPs and InDels throughout the assembled genome to estimate the sequence diversity of the parents. We identified genomic intervals located in each chromosome that had reduced numbers of SNPs and InDels, which may indicate that these regions have lower sequence diversity (data not shown). In the regions of low SNPs and InDels, we identified ∼50 and ∼100 markers that had an average recombination rate of ∼0.6 and ∼0.9 cM/Mb respectively, which was similar to the recombination suppression regions rate (0.4 cM/Mb) and lower than the whole genome recombination rate (2.3 cM/Mb). These markers occurred in recombination suppression regions, which mean these low-sequence-diversity regions overlapped with the low-recombination regions identified in the genetic map, indicating they could be in the pericentromeric regions [34].

High recombination in gene-dense regions and proximity to telomeres are more important for breeding, because meiotic recombination was frequent in these regions, where crossover and gene conversion can occur, leading to potentially useful genetic variation. Recombination rates that increase with relative distance from centromeres have been observed in many organisms including maize, wheat and barley [35], [36], [37]. The high recombination regions can produce large genetic distance in limited physical distance, such as across the *a1-yz1* region in maize, the ratio of genetic-to-physical distance varied from 6.9 cM/Mb to 11 cM/Mb [38]. The limited genomic resources available in previous genetic mapping studies of watermelon [2], [3], [4], [10] prohibited whole genome recombination rate analysis. In our research, each marker was designed from a non-repeat scaffold sequence, which can theoretically permit detection of crossovers throughout the whole genome. The regions with high SNP and InDel density were enriched in recombinationally active chromosome ends, indicating they are in the telomere and gene-rich regions [39].

### Future research perspective

In this research, we successfully anchored 234 scaffolds (93.5% of the assembled 353 Mb sequences) using a high resolution map constructed from a population consisting of 103 recombinant inbred lines (RILs) and 228.3 Mb (65%) of the assembled sequences were oriented. Maps for several crop species have been recently published and the percentages of anchored and oriented assembly sequences are: cacao (67% anchored, 50% oriented) [40]; apple (88%, 66%) [41]; grape (69%, 61%) [42]; strawberry (∼94% anchored) [43]; soybean (97% anchored) [44]; and cucumber (72.8% anchored) [18]. By comparison, the percent of anchored scaffolds (93.5%) in our research was at very high level, but the percent of oriented (65%) sequences was not high, but similar to the highest extent of orientation reported in other crop species. The factors that limit successful orientation and ordering of scaffolds are mainly map density and population size. For non-ordered scaffolds with only one genetic marker or located in a genomic region with low recombination frequency, their positions on the map may be uncertain with the adjacent scaffolds. A higher resolution genetic map using a larger genetic population will be needed to order and orient the remaining sequences. For example, a higher resolution single nucleotide polymorphism (SNP) map based on population of 444 soybean RILs was used to orient 151 scaffolds [44], which were previously un-oriented in the Soybean Consensus Map 4.0 that was based on a population of 89 RILs. In the future, other molecular markers such as SNPs present in a larger mapping population will need to be used in order to construct a higher density watermelon map.

Because a definitive karyotype such as a karyotype based on C-banding or FISH, does not exist for watermelon, the identification of the eleven morphologically indistinct watermelon chromosomes remains impossible. Here, we integrated 11 linkage groups into 11 chromosomes and assigned one of 11 single-copy BACs to each chromosome. Consequently, these BACs can be used for assigning linkage groups to chromosomes, but without additional cytological data the chromosomes remain morphologically indistinct. Future cytological research to identify each of the eleven morphologically indistinct chromosomes is necessary.

The polymorphic rate of SSR markers (41.9%) of the distant watermelon lines was relatively higher than previously reported (26%). This may be due to the small number of previously reported SSRs (43) [4], which cannot reflect the real polymorphic rate of whole genome sequence. To some extent, the high polymorphic rate between the wild watermelon and cultivars may be a little lower in the cultivars. However, the high density watermelon genetic map, which reflects integration of the whole genome scaffold sequences, will be useful for positional cloning of important genes, identification of QTLs, and marker assisted selection (MAS). The work reported here should greatly benefit watermelon breeding.

## Supporting Information

Table S1 SSR, InDel and SV markers used to construct the saturated genetic map of watermelon for scaffold anchoring. Their chromosome, bin position, genetic distance (cM), marker name, repeat motif, scaffold name, primer start, primer end, production size from 97103 and primer sequences are listed.
(XLS)

Table S2 Selected BACs for assigning linkage groups to chromosomes. Marker name, genetic distance, corresponding scaffolds, unique signal BACs and BACs/45S/5S for assigning LGs to chromosomes are listed.
(XLS)
